# Tracking the emergence of a novel genotype of *Decapod hepanhamaparvovirus* in shrimp using laser microdissection and next generation sequencing

**DOI:** 10.1371/journal.pone.0311592

**Published:** 2024-10-10

**Authors:** Roberto Cruz-Flores, Arun K. Dhar

**Affiliations:** 1 Aquaculture Pathology Laboratory, School of Animal and Comparative Biomedical Sciences, The University of Arizona, Tucson, Arizona, United States of America; 2 Centro de Investigación Científica y de Educación Superior de Ensenada (CICESE), Ensenada, Baja California, México; University of Basrah, IRAQ

## Abstract

The prevalence of hepatopancreatic diseases in cultured shrimp has increased in recent years. *Decapod Hepanhamaparvovirus* 1 (DHPV) infection was identified by histology in samples that could not be detected by PCR-based assay for this virus. Employing Laser Microdissection (LMD), we dissected cells containing intranuclear inclusion bodies pathognomonic for DHPV infection from histological sections. Whole Genome Amplification and NGS were used to generate five complete genomes of the novel DHPV isolate that showed identities ranging from 77% to 98% to previously reported isolates. Phylogenetic analyses revealed the DHPV isolate represents a novel genotype, Genotype V. We developed PCR and *in situ* hybridization methods tailored for the specific detection of this genotype. Our approach of combining LMD with NGS opens avenues for rapid identification of emerging viral pathogens and retrospective studies to understand origin and evolution of viruses showcasing the transformative potential of the innovative approach used in this study.

## Introduction

Accurate and sensitive pathogen detection is only achievable through the combination of complementary diagnostic techniques. This becomes relevant when dealing with viral pathogens that have very high mutations rates such as viruses with ssRNA and ssDNA genomes [1, 2]. *Decapod penstylhamaparvovirus* 1 commonly known as Infectious Hypodermal and Hematopoietic Necrosis Virus (IHHNV) is a shrimp parvovirus (family *Parvoviridae*) that has been found to poses a very high mutation rate comparable to those of ssRNA [2, 3]. A close relative of IHHNV within the sub-family *Hamaparvovirinae* is *Decapod hepanhamaparvovirus* 1 (DHPV) commonly known as Hepatopancreatic parvovirus (HPV) [4]. The genetic variation of DHPV strains is considerable and a genetic drift of 8–15% has been reported by [5]. It is plausible to consider that the substantial genetic diversity observed among DHPV strains may be attributed to an exceptionally elevated mutation rate, akin to that of IHHNV.

Single-stranded DNA viruses do not possess proof-reading activity as the DNA replicase does not have a complement strand to compare against [5]. This of course leads to higher mutation rates and, most importantly, from a disease diagnostic perspective it directly impacts the accuracy of PCR and qPCR based diagnostic assays. In recent years, DHPV diagnostics by PCR/qPCR methodologies has been particularly challenging. The emergence of several novel DHPV (GenBank: ON872187.1) strains from Korea has exemplified this challenge as PCR-based methodologies recommended by the World Organization for Animal Health (WOAH, Paris, France) failed to detect this particular viral strain [6]. Several research groups have encountered this problem and sensitive universal semi-nest PCR methodologies have been developed for the detection of several DHPV strains [7].

While a substantial genomic variation exists within the *Hepanhamaparvovirus* genus, one enduring aspect that unifies this pathogenic group is the consistent pathological manifestation of the virus at a histological level. This positions histological examination, for the current time, as the gold standard for diagnosis for DHPV. The hepatopancreas is the principal target tissue of this virus. DHPV’s infection progression entails the infiltration of tubule epithelial cells, culminating in the development of prominent intranuclear basophilic inclusion bodies within the E- and F-cells, predominantly concentrated along the distal section of the hepatopancreatic tubules [8].

**Fig 1:**
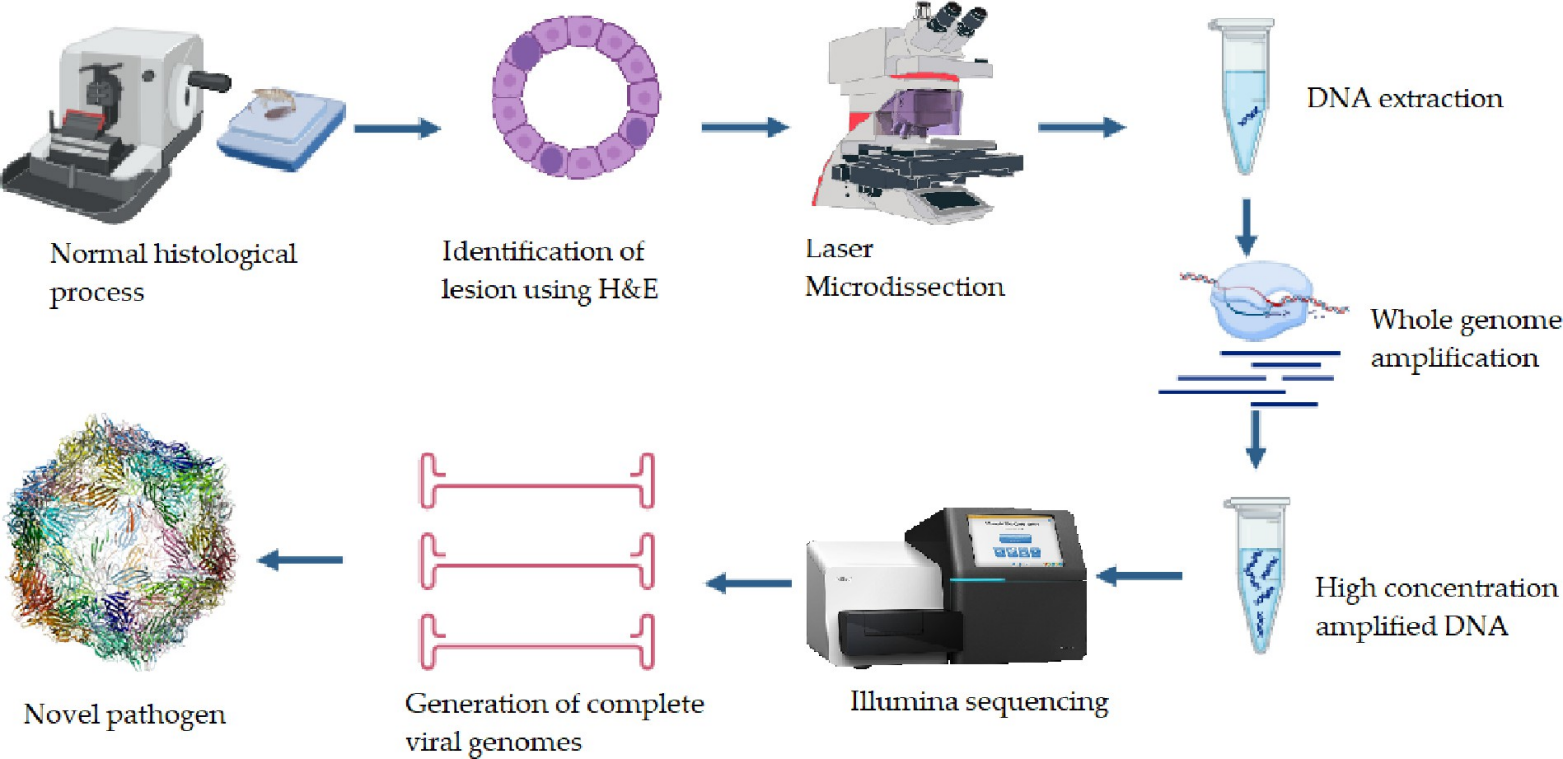
Pathogen discovery pipeline using molecular histology. Our innovative pathogen discovery pipeline leverages the power of molecular histology, starting with the histological processing of the tissue of interest. Lesions or alterations are identified with precision and selected using a Laser Microdissection Microscope. From these, individual cells or a small cluster of cells are isolated for nucleic acid extraction. Given the minute quantity of nucleic acids, we employ whole genome amplification to increase their concentration. The amplified DNA is then sequenced using an Illumina sequencer. Bioinformatic analyses are conducted to assemble the viral genome, leading to the discovery of novel pathogens or new strains of existing ones.

In 2021, Aquaculture Pathology Laboratory in the University of Arizona, a Reference Laboratory of the WOAH for Crustacean Diseases (https://www.woah.org/en/what-we-offer/expertise-network/reference-laboratories/#ui-id-3) received diagnostic cases from a country in Latin America that presented the distinctive inclusion bodies that are pathognomonic of DHPV infection. However, PCR-based diagnostics failed to detect the virus in these samples. In this study, we employed a Laser Microdissection (LMD) to selectively microdissect infected cells containing inclusion bodies, reducing the presence of apparently healthy cells to ultimately minimize the host-related nucleic acids in the genomic DNA to facilitate subsequent bioinformatics analysis. Given the scant amount of nucleic acids within the microdissected cells, we conducted Whole Genome Amplification (WGA) to obtain a sufficient DNA quantity for subsequent Next Generation Sequencing (NGS). We report here a methodology for sequencing the complete genome of a viral agent from a limited number of cells derived from formalin-fixed paraffin-embedded (FFPE) tissue, including the complete genome of a novel genotype of DHPV (Fig 1).

## Material and methods

### Sample origin and diagnostic evaluation

Davidson fixed shrimp (*Penaeus vannamei*) samples were received from a country in Latin America in 2021. Fixed samples were processed in a tissue processor, embedding in paraffin and sectioned (5 μm thick) using a standard methodology [9]. Tissue sections were stained with H&E following standard procedures. Histological slides were examined using a bright field light microscope.

Total genomic DNA was isolated from paraffin embedded tissue sections using a Arcturus®Pico Pure®DNA extraction kit and DHPV detection carried out following two published protocols [7, 10].

### Laser Capture Microdissection, DNA extraction and Whole Genome Amplification

Five FFPE tissue blocks of shrimp (case 21–269) that displayed the typical intranuclear inclusion bodies formed by DHPV were selected for LMD. Deparaffinized 5 μm sections were mounted on PEN membrane glass slides (Life Technologies) and stained with Arcturus® Paradise® PLUS Reagent System [11]. The slides were labeled as 21-269-1, 21-269-3/1, 21-269-3/2, 21-269-4/1, and 21-2694/2. DHPV inclusion bodies were dissected using an LMD7-Laser Microdissection Microscope (Leica Microsystems™). DNA was extracted using Arcturus®Pico Pure®DNA extraction kit. Considering the extremely low quantity of DNA derived from few cells, the DNA was amplified using a whole genome amplification method and the SeqPlex DNA Amplification Kit (Sigma-Aldrich) following the manufacturers recommendations. The WGA DNA was sent to OmegaBioservices, Norcross, GA. Library for the DNA samples were generated at OmegaBioservices using the Library Kit, KAPA Hyper prep for WGS (Roche). The samples were sequenced using an Illumina HiSeq 2500 System (PE 2X150PE).

### Bioinformatics analysis

The DNA reads were paired and duplicate reads were removed using the Dedupe plugin in Geneious Prime version 2023 [12]. DNA reads from the DHPV infected shrimp were checked for quality and trimmed using the BBDuk plung in and were *De Novo* assembled using the Geneious assembler with the default parameters with one modification. The program was set to circularized contings if ends matched. The mean coverage of each based was calculated. The contigs generated from the *De Novo* assembly were annotated using BLASTn and Geneious Prime [12, 13]. The complete genomes, NS1, NS2 and the VP genes were compared with BLASTn and BLASTp [13]. The complete DHPV genomes reconstructed from LMD derived DNA (GenBank accessions: PP417729, PP417730, PP417731 and PP417731) were submitted to GenBank.

### Identity confirmation by PCR and in situ hybridization

Two primers located in the capsid region were designed utilizing Geneious Prime (S1 Fig). The primer sequence and location are shown in Table 1. Furthermore, both the WOAH reference primers and the newly designed primers were aligned with the reference sequence of DHPV (NC_014357) and the novel DHPV genotype (PP417729) to identify and visualize any mismatches within the primer regions, thereby ensuring the specificity of the primers (S1 Fig). DNA from the five blocks was isolated using a commercially available FFPE DNA Purification Kit (NORGEN BIOTEK CORP). The extraction protocol closely followed the manufacturer’s recommendations, with minor modifications. During the deparaffinization step, the number of xylene washes were doubled, and the resulting pellet was air-dried for 20 minutes. Moreover, in the lysate preparation stage, the incubation at 90°C was extended from 1 hour to 1 hour and 15 minutes. Each sample yielded two elution.

**Table 1:** Primers designed for the specific detection of a novel genotype of *Decapod hepanhamaparvovirus* 1 by conventional PCR from FFPE derived DNA.

Primer name	Location on the PP417729 genome	Primer sequence (5´ to 3´)	Product size
VP 1F	4,554–4,573	ACGACAGGTTGACATGGACC	147 bp
VP 1R	4,700–4,681	CCAACTCGAGGTTCCCCATC	
VP 2F	3,851–3,870	CAGTTGGGACGTGACAGTGA	122 bp
VP 2R	3,972–3953	ATGGCTGTTGTTGCTGTCCT	

Each PCR amplification was conducted in a total volume of 25 μl containing 1 μl of template DNA (50–100 ng/ μl), 12.5 μl of DreamTaq Hot Start Green PCR Master Mix (ThermoFisher) and 350 nM of each primer pair targeting the viral capsid gene, VP (VP 1F/1R and VP 2F/2R). The PCR conditions consisted of an initial denaturation at 95°C for 2 min, followed by 45 cycles at 95°C for 10 s, 60°C for 10 s and 72°C for 10 s with a final elongation step at 72°C for 2 min. The PCR products were run on a 2% agarose gel and were visualized on a GelDoc XR + (Bio-Rad). The amplicons were sequenced at the University of Arizona Genetics Core.

For *in situ* hybridization, all sections were dried onto positively charged microscopic slides and *in situ* hybridization was carried out following the protocols described by [14, 15] with an equivolume mixture of four primers VP 1F, VP 1R, VP 2F and VP 2R. These primers were tailed at 3′-end with digoxigenin-11-dUTP (Integrated DNA Technologies®, San Diego, CA). After deparaffinization, hydration, proteinase K digestion, and pre-hybridization, the sections were overlaid with 500 μL of hybridization solution containing DIG-labeled primers (100 fmol). The slides were placed on a heated surface at 90˚C for 10 min and hybridized overnight at 50˚C. Final detection was performed with an anti-digoxigenin antibody conjugated to alkaline phosphatase (Roche), which was visualized using nitro blue tetrazolium and 5-bromo-4-chloro-3-indolyl phosphate [16].

### Phylogenetic analysis

To investigate the evolutionary relationship within the DHPV genomes and other *Hepanhamaparvovirus*, we conducted a series of phylogenetic analyses. The GenBank accession numbers of the utilized genomes are: JN082231, GU371276, EU346369, ON872187, AY008257, DQ002873, EU588991, FJ410797, EU2475528, PP417729, PP417730, PP417731, PP417731, and AF273215 (*Decapod penstylhamaparvovirus* 1 outgroup). For the phylogenetic analysis, two trees were constructed, one tree utilized the full genome nucleotide sequence, and the second tree utilized the complete coding region of the capsid protein gene. To obtain the capsid gene sequence, the corresponding annotation was extracted in Geneious prime and translated. The process of constructing the phylogenetic trees began with the alignment of the sequences using the MUSCLE algorithm in Geneious Prime [12, 17]. The evolutionary lineage was deduced by applying the Neighbor-Joining method, while employing the p-distance model [18, 19]. The percentage of replicate trees in which the associated taxa clustered together in the bootstrap test (1000 replicates) are shown next to the branches [20]. The tree is drawn to scale, with branch lengths in the same units as those of the evolutionary distances used to infer the phylogenetic tree [21].

## Results

### Detection of DHPV using the WOAH recommended PCR and histological examination

**Fig 2:**
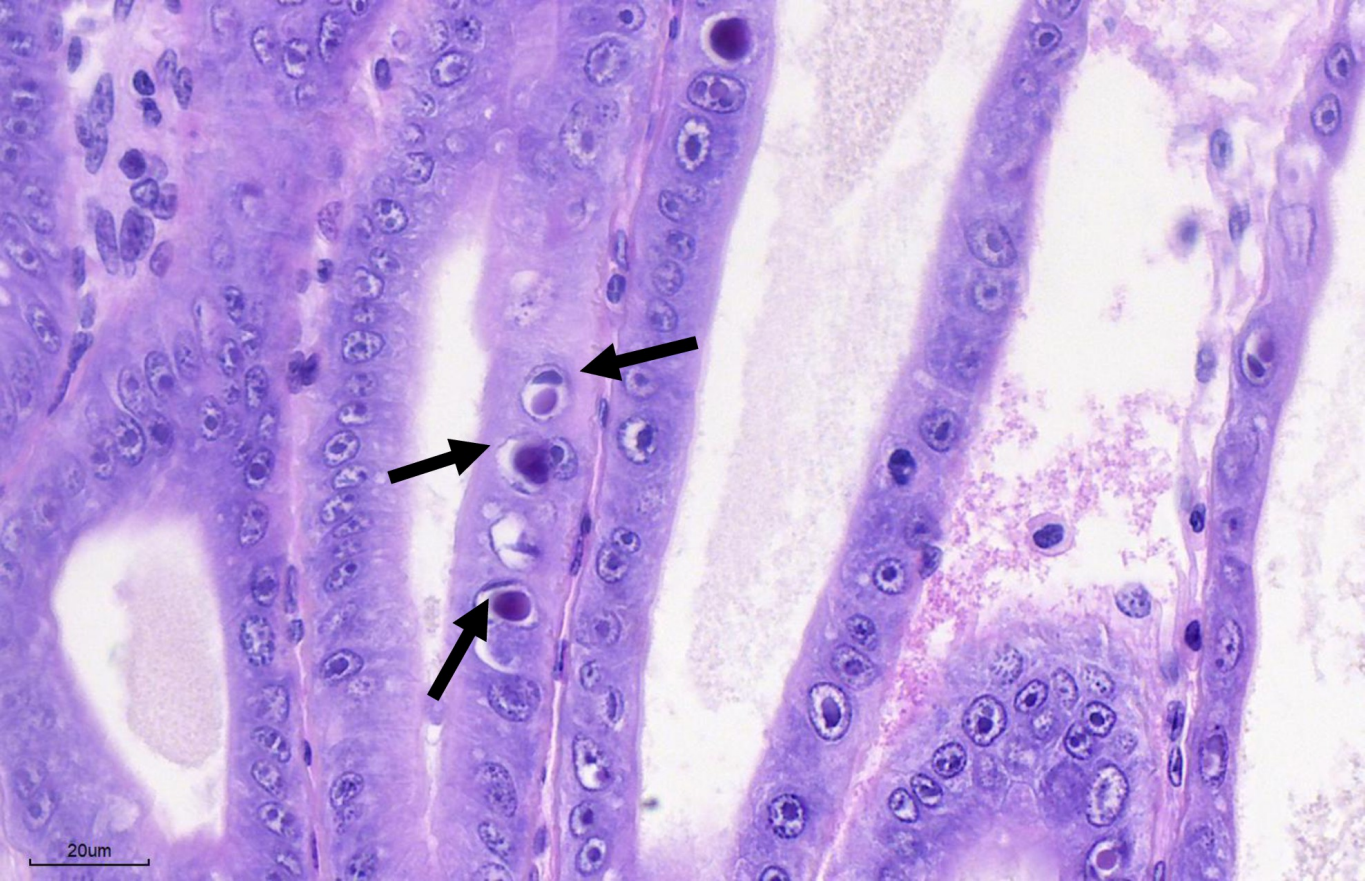
Distal tubules of the hepatopancreas of *Penaeus vannamei* from case 21–269 showing the typical basophilic intranuclear inclusion bodies (black arrows) caused by DHPV.

Using H&E histology, the diagnostic cases displayed the presence of prominent intranuclear basophilic inclusion bodies within the E- and F-cells. The inclusion bodies were concentrated along the distal end of the hepatopancreatic tubules (Fig 2). However, following a published protocol for DHPV detection [10], the virus could not be detected in these samples. The sequence alignment of the WOAH reference primers revealed multiple mismatches in the reverse primer binding region (S1 Fig), suggesting that these primers may not be compatible. In addition, the semi-nested PCR methodology recently described by [7] also yielded negative results.

### Laser Microdissection of the intranuclear inclusion bodies formed by DHPV

**Fig 3:**
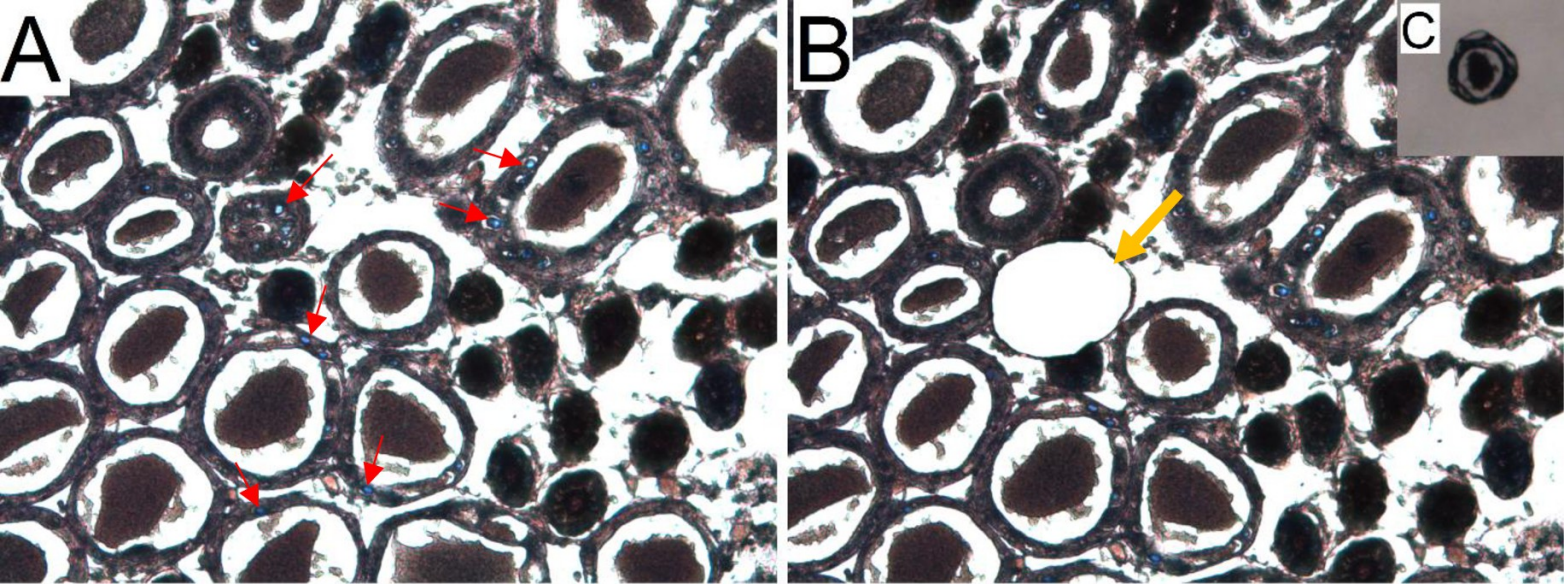
*Decapod hepanhamaparvovirus* intranuclear inclusion bodies stained with Arcturus® Paradise® PLUS Reagent. The slides are not mounted (no cover slip). (A) A tissue section prior to microdissection. The intranuclear inclusion bodies formed by DHPV stained bright blue to azure. Some inclusions are shown by the red arrows. (B) Same tissue section that displays tubule epithelial cells with heavily infected cells were dissected (orange arrow). (C) A dissected tubule in the cap of a 0.6 ml tube ready for DNA extraction.

The intranuclear inclusion bodies formed by DHPV stained bright blue to azure with the Arcturus® Paradise® PLUS stain and were easily differentiated from the grayish to brown host tissue (Fig 3A). Utilizing a Laser Microdissection Microscope hepatopancreatic tubule cells heavily infected with candidate DHPV were Microdissected and taken for isolation of total genomic DNA (Fig 3B and 3C).

### Analysis of the novel DHPV isolate

Next Generation Illumina sequencing of the five samples derived from LDM DNA yielded 13,477,364, 7,672,328, 7,422,530, 5, 356,582 and 7,199,404 cleaned PE reads for samples 21-269-1 (GenBank accession: PP417729), 21-269-3/1 (GenBank accession: PP417732), 21-269-3/2, 21-269-4/1 (GenBank accession: PP417731) and 21-2694/2 (GenBank accession: PP417730), respectively. The complete genome length for each sample is 6,195 nt, 6,196 nt, 6,195, 6,195nt and 6,197 nt for samples 21-269-1, 21-269-3/1, 21-269-3/2, 21-269-4/1, and 21-2694/2, respectively. The viral sequences identified within the assembled contigs comprised 17.96%, 5.21%, 17.51%, 18.19%, and 19.11% of the total reads obtained from samples 21-269-1, 21-269-3/1, 21-269-3/2, 21-269-4/1, and 21-269-4/2, respectively. The five generated genomes were almost identical with an identity that ranged from 99.96–100% (Table 2).

**Table 2:** Nucleotide identity (%) between the five genomes generated from samples 21-269-1, 21-269-3/1, 21-269-3/2, 21-269-4/1, and 21-2694/2. A high sequence identity (99.96–100%) was observed between the five samples.

	21-269-1	21-269-3/1	21-269-3/2	21-269-4/1	21-2694/2
**21-269-1**		100	100	99.98	99.98
**21-269-3/1**	100		100	99.98	99.96
**21-269-3/2**	100	100		99.98	99.98
**21-269-4/1**	99.98	99.98	99.98		100
**21-2694/2**	99.98	99.96	99.98	100	

Sequence analysis of the complete genome of the novel DHPV isolate using sequence 21-2694/2 showed a 98.53% identity at a whole genome level with the recent Korean decapod hepandensovirus isolate Pv/2021/21-044B (Accession: ON872187.1). Furthermore, amino acid (aa) sequence analysis of the NS1, NS2 and VP genes showed a 97.57%, 99.29% and 96.82% identity to Korean decapod hepandensovirus NS1 gene (Accession: WOJ46325.1), Hepandensovirus sp. NS2 gene (Accession: WLG15887.1) and Hepandensovirus sp. structural protein (Accession: UXK32600.1) respectively, as seen in Table 3.

**Table 3:** Nucleotide and amino acid sequence identity of the complete genome, NS1, NS2 and VP genes of the novel genotype of *Decapod hepanhamaparvovirus* from Latin America.

Virus	Accession	Query Cover (%)	E-value	Percent Identity (%)
Whole Genome				
Korean decapod hepandensovirus isolate Pv/2021/21-0044B	ON872187.1	99	0.0	98.53
Hepandensovirus sp strain GJ2022	OQ857568.1	95	0.0	88.22
*Penaeus chinensis* hepandensovirus	AY008257.2	92	0.0	77.35
**NS1**				
Nonstructural protein 1 (Korean decapod hepandensovirus)	WOJ46325.1	100	0.0	97.57
Nonstructural protein 1 (Hepandensovirus sp)	WLG15885.1	100	0.0	97.40
Nonstructural protein 1 (Penaeus chinensis hepandensovirus)	ABY60414.1	99	0.0	87.65
**NS2**				
Nonstructural protein 2 (Hepandensovirus sp)	WLG1588.7.1	100	0.0	99.29
Nonstructural protein 2 (Hepandensovirus sp)	WLG15888.1	100	0.0	85.31
Nonstructural protein 2 (Penaeus monodon hepandensovirus)	YP271914.1	100	0.0	67.60
**VP**				
Structural protein (Hepandensovirus sp)	UXK32600.1	99	0.0	96.82
Capsid protein (Korean decapod hepandensovirus)	WOJ46326.1	99	0.0	96.40
Structural protein (Hepandensovirus 4)	YP_00230847	97	0.0	62.27

### Confirmation of DHPV presence by PCR and in situ hybridization

**Fig 4:**
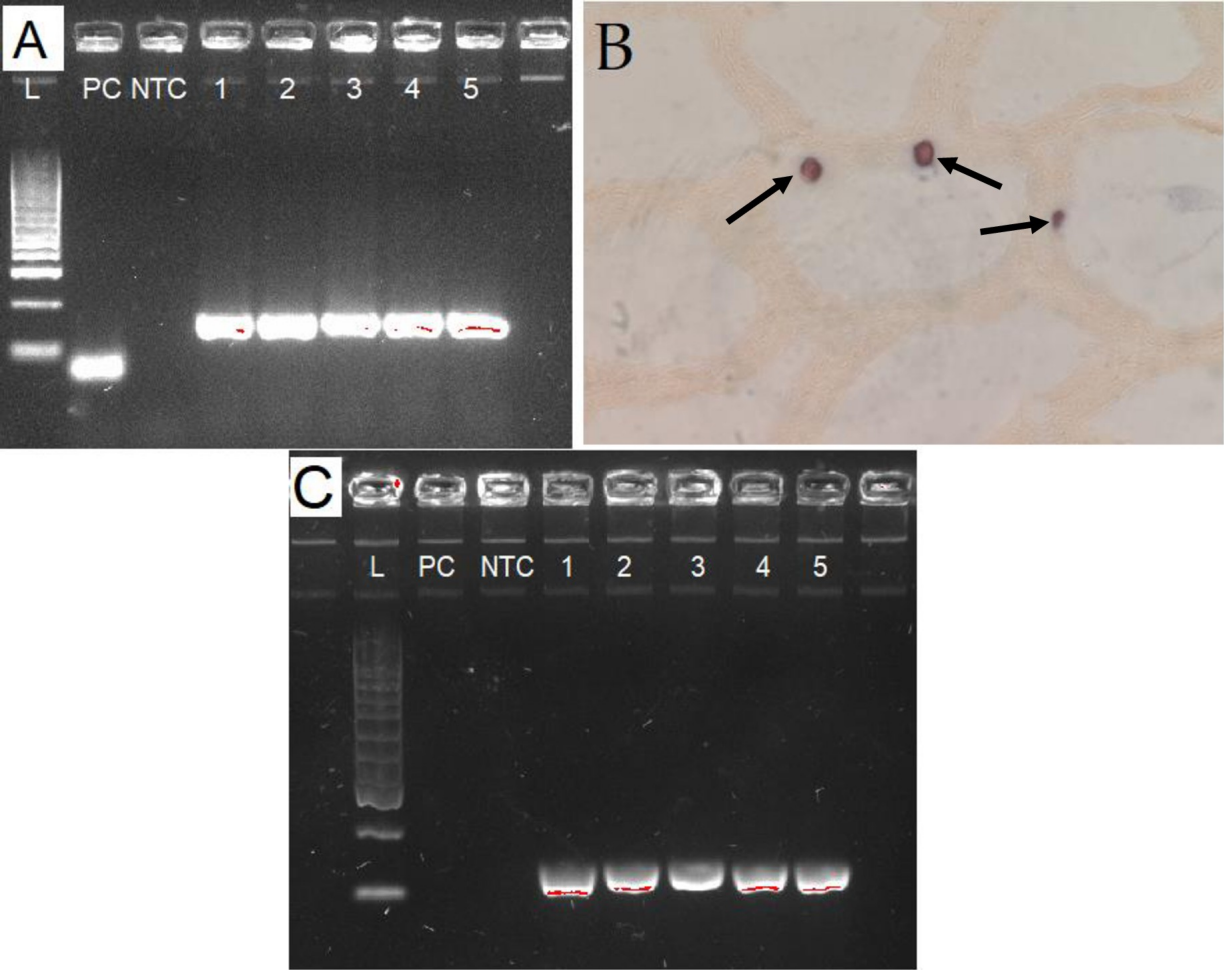
Confirmation of presence of the novel genotype of *Decapod hepanhamaparvovirus* from Latin America by PCR and ISH. (A and C) Gel electrophoresis of PCR products generated using primers VP 1F/1R and VP 2F/2R from samples 1 (269–1), 2 (21-269-3/1), 3 (21-269-3/2), 4 (21-269-4/1) and 5 (21-2694/2). The positive control (PC) was represented by plasmid DNA isolated from a DHPV clone routinely used for the detection of the virus following the WOAH recommended protocol. NCT = No template control. PCR products of the expected size of ~147 bp and ~122 bp for panel A and C respectively are observed in all five samples while the PC did not provide any amplicon. (B) Detection of a novel DHPV isolate from Latin America using an *in situ* hybridization using DIG-labelled probe. A dark brown precipitate indicating the presence of DHPV inclusion bodies is shown by red arrows.

Two primer pairs were designed based on the capsid protein gene of the novel DHPV. These primers amplified a 147 bp amplicon with the five samples tested and did not react with the DHPV DNA that is routinely used as a positive control in PCR amplification following the WOAH protocol (Fig 4). Furthermore, when these primers were labelled with DIG and used for *in situ* hybridization, a strong reaction was observed in the inclusion bodies formed by DHPV in all five samples (21-269-1, 21-269-3/1, 21-269-3/2, 21-269-4/1, and 21-2694/2) (Fig 4).

### Phylogenetic analysis

**Fig 5:**
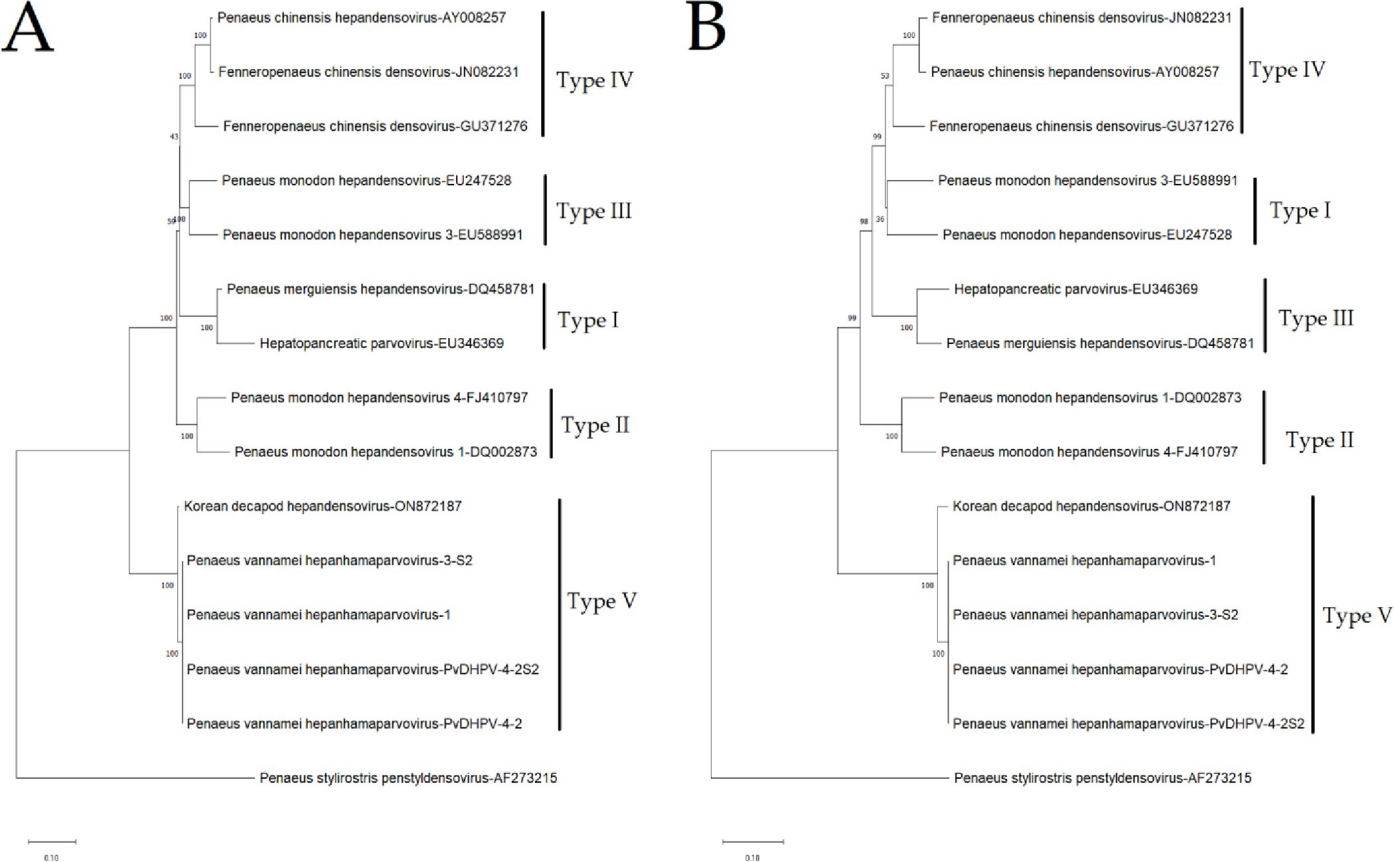
The evolutionary relationship of the newly identified DHPV isolate from Latin America to previously known genotypes of *Decapod hepanhamaparvovirus* 1 based on nucleotide and amino acid sequences (A) Whole genome phylogeny of reference DHPV genotypes clustering into the well-recognized genotypes I, II, III and IV. The novel DHPV isolate from Latin America forms a well-supported cluster with a new Korean isolate. We have termed this group as genotype V. (B) Capsid protein phylogeny of the same DHPV genotypes. An identical topology was observed utilizing the capsid protein sequence with the formation of five genotypes representing types I, II, III, IV and V.

There are four widely recognized genotypes of *Decapod hepanhamaparvovirus* 1. The novel genotype from this study did not cluster with any of the known genotype and it formed a highly supported cluster with the recently described DHPV isolate from Korea (ON872187.1) in both phylogenies that are based on the complete genome sequence and amino acid sequence of the capsid protein (Fig 5).

## Discussion

Laser microdissection and Laser Capture Microdissection were developed within the realm of solid tissue analysis in addressing the inherent complexity and heterogeneity of these structures composed of various morphologically and functionally distinct cell types. Consequently, when conducting whole-tissue analysis, the resulting outcomes are often dictated by the prevalent cell type, potentially obscuring biologically significant changes present in specific cell subsets or a minority of cells. The advent of advanced yet user-friendly laser-based methodologies has facilitated studies that seamlessly integrate microscope-based morphological analysis with a wide array of potent molecular technologies [22, 23]. LMD has advanced the study of host-microorganism interactions and several studies have characterized the host response to commensal or pathogenic bacterial infections in the intestine [24], stomach [25], lung [26] and bladder [27] at a gene expression level [28] in terrestrial animals and human. In addition, LMD has been recently applied to analyze the spatial architecture of the bacterial microbiota of mucosal tissues [29–31]. While the use of LMD/LCM coupled with whole genome amplification (WGA) techniques has been postulated as a powerful tool to study low intensity and/or intracellular pathogens [32, 33], to date no studies have been successful at sequencing the complete genome of a viral pathogen derived from LMD cells. To our knowledge, this is the first study that has successfully sequenced the complete genome of a virus from LMD derived DNA that had been subjected to WGA. Previously, the complete genome of white spot syndrome virus had been reconstructed from FFPE blocks without LMD and only a small portion (~1%) of the total reads were associated with the virus [34]. In this study, up to 19% of the total read were associated with the pathogen. This represents a significant augmentation of the total virus associated read which has a direct impact in facilitating and reducing time needed for bioinformatics analysis. This methodology could be especially useful when the infection intensity is very low or limited to a few cells. Furthermore, it could allow us to perform retrospective studies on uncharacterized viruses at a whole genome level from histological samples that were deemed inadequate for molecular studies due to the limited number of viral inclusions and/or alterations.

In recent years, there has been an increase in the prevalence of hepatopancreatic diseases in cultured shrimp populations (Dhar *et al*., *unpublished)*. Globally in the last three years, *Enterocytozoon hepatopenaei* (EHP), *Penaeus monodon* nudivirus (PmNV), *Penaeus vannamei* Solinvivirus (PvSV), Necrotizing hepatopancreatitis bacterium (NHP-B), various *Vibro* sp. causing acute hepatopancreatic necrosis disease (AHPND) and DHPV have been frequently detected in the tubule epithelial cells of hepatopancreas by histological and/or molecular surveys [6, 35–40]. Among these infectious agents, DHPV has been detected with increasing frequency in our laboratory. Interestingly, we have observed divergence not just at the genomic level but also some DHPV variants show variation in their tissue tropism. In one diagnostic case from 2021, we detected shrimp that presented with typical DHPV lesions in the hepatopancreas but did not provide positive result with any of the known molecular methods recommended by the WOAH or the universal PCR method recently reported by [7]. This case presented a unique opportunity for us to explore if LMD could be used to selectively dissect cells displaying pathognomonic lesions of DHPV. Employing a combination of LMD and WGA amplification techniques, we were able to successfully sequence and characterize the complete viral genome of this unique DHPV strain. Furthermore, we developed specific PCR methods tailored to detect this particular genotype of DHPV, which did not respond to previously reported methods. A significant challenge that remains with this pathogen is the development of a universal detection method capable of identifying all DHPV genotypes.

Review of the literature showed DHPV isolates infecting various shrimp species across diverse geographical regions consistently represent significant genetic diversity at both the nucleotide and amino acid levels. [10, 41]. The highest level of sequence similarity was found in the amino acid sequence of the NS1 gene (97.1–99.8%) [41] which is consistent with the present study where similarity ranging from 87–97% was recorded. In contrast, the amino acid VP sequence is known to be the most variable of the DHPV genes with a mean genetic distance of 24% among isolates [10, 39]. Our study was also in accordance with previous studies where amino acids sequence identity ranged from 62–96%. Overall, the DHPV isolate from Latin America showed the highest nucleotide identity (98%) with a recently reported Korean isolate [39].

Phylogenetic relationships between geographically different DHPV genotypes has traditionally been studied employing the amino acid sequences of the VP1 and NS1 genes [10, 39, 41]. Traditionally, phylogenetic clustering indicates the existence of three distinct DHPV genotypes [10, 42]. Type I includes isolates from Korea, Madagascar, and Tanzania; Type II includes isolates from Thailand and Indonesia, while Type III contains isolates from Australia and New Caledonia [10, 42]. More recently, utilizing an increased number of sequences a fourth well-supported genotype (Type IV) was identified that contained Korean and Chinese isolates [3, 41]. However, in a recently published article Kim et al., (2024) reported DHPV isolates from Madagascar, Tanzania, Korea and Chinese Isolates as a single genotype (Genotype I) although these isolates formed two distinct clades (see Fig 3A and 3B in Kim et al., 2024). The study of Kim et al., [39] also proposed the emergence of a new genotype. In this study, we observed a similar topology to what was previously reported by Dhar et al., [3, 41]where four genotypes are identified. Furthermore, in accordance with Kim et al., [39] the Korean isolate forms an additional well supported cluster with the newly reported Latin American isolates. Considering the high sequence divergence at a nucleotide and amino acid level and that these isolates are the only ones, for the moment, reported from *P*. *vannamei* we propose the formation of a Type V genotype.

In summary, our study is a showcase of an innovating approach for pathogen discovery leveraging LMD coupled with WGA to sequence a viral genome from a restricted number of infected cells isolated from Davidson fixed paraffin embedded histology tissue samples. This innovative strategy substantially augments the yield of virus-associated sequences, streamlining subsequent bioinformatics analyses and significantly curtailing the time required for comprehensive characterization. Furthermore, the identification of a novel DHPV isolate originating from Latin America unveils a distinctive genetic profile, diverging markedly from previously documented DHPV genotypes. Our findings propose the classification of this isolate into a newly proposed genotype, denoted as ’Type V’. This novel approach not only holds promise for expediting pathogen discovery but also enables retrospective investigations into previously unexplored viruses at a whole-genome level, utilizing histological archives. The Aquaculture Pathology Laboratory, a Reference Laboratory of Crustacean Diseases of the World Organization for Animal health (Paris, France), has an archives of over 110,000 histological blocks dating back to mid-70’s originating from many countries around the world. These histological tissue archives are valuable biological materials to study viral evolution, epidemiology, and pathogenesis, thus underscoring the transformative potential of the finding presented in this paper.

## Supporting information

S1 Fig Alignment of primer regions, comparing the WOAH reference primers (Panels A and B) with the two novel primer pairs designed for this study (Panels C and D).
(ODT)

S1 File (ZIP)
