# Supplementary figures and images for: Tracking the emergence of a novel genotype of Decapod hepanhamaparvovirus in shrimp using laser microdissection and next generation sequencing
Source: PLoS One. 2024 Oct 10;19(10):e0311592. doi: 10.1371/journal.pone.0311592 (PMC11469598; doi:10.1371/journal.pone.0311592)

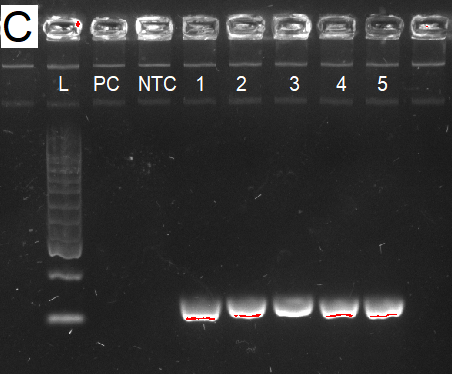

Supplement: S1 File — (ZIP) [file pone.0311592.s002.zip › Figure 3, Panel C- ORIGINAL, SEPT 13, 2023.png]

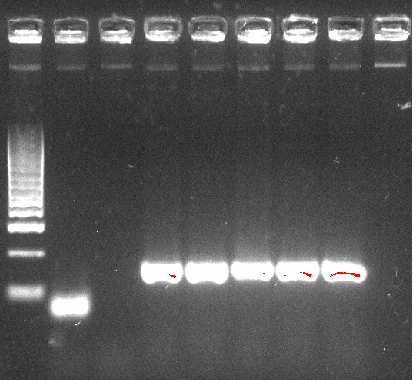

Supplement: S1 File — (ZIP) [file pone.0311592.s002.zip › Figure 3, Panel A - ORIGINAL, SEPT 13, 2023.png]
