# Supplementary material for: Tracking the emergence of a novel genotype of Decapod hepanhamaparvovirus in shrimp using laser microdissection and next generation sequencing
Source: PLoS One. 2024 Oct 10;19(10):e0311592. doi: 10.1371/journal.pone.0311592 (PMC11469598; doi:10.1371/journal.pone.0311592)
Supplement: Supplementary file 4 [file pone.0311592.s001.odt]

Supplementary data for: Tracking the emergence of a novel genotype of Decapod hepanhamaparvovirus in shrimp using laser microdissection and next generation sequencing

Short title: Laser microdissection for pathogen discovery

Roberto Cruz-Flores1,2 and Arun K. Dhar1*

1Aquaculture Pathology Laboratory, School of Animal and Comparative Biomedical Sciences, The University of Arizona, Tucson, Arizona.

2Centro de Investigación Científica y de Educación Superior de Ensenada (CICESE), Carretera Ensenada-Tijuana No. 3918, Zona Playitas, 22860 Ensenada, Baja California, México

*Corresponding author: Arun K. Dhar, E-mail: [adhar@arizona.edu](mailto:adhar@arizona.edu) 

Texto, Aplicación

Descripción generada automáticamente

Supplementary Figure 1. Alignment of primer regions, comparing the WOAH reference primers (Panels A and B) with the two novel primer pairs designed for this study (Panels C and D). In all panels, the sequence at the top corresponds to the DHPV reference genome (NC_014357), while the sequence at the bottom represents one of the newly identified DHPV genomes (PP417729). Nucleotides that differ between the sequences are highlighted. (A) Primer binding region of the WOAH-recommended primer 2F for DHPV detection, showing no observed discrepancies. (B) Primer binding region of the WOAH-recommended primer 2R for DHPV detection, displaying five mismatches. (C) Primer binding regions for the newly designed VP1F and VP1R primers, showing five mismatches in the VP1F region and six mismatches in the VP1R region. (D) Primer binding regions for the newly designed VP2F and VP2R primers, with five mismatches in the VP2F region and six mismatches in the VP2R region.
